# A comparison of radiographic and clinical outcomes of anterior lumbar interbody fusion performed with either a cellular bone allograft containing multipotent adult progenitor cells or recombinant human bone morphogenetic protein-2

**DOI:** 10.1186/s13018-017-0618-8

**Published:** 2017-08-25

**Authors:** Daniel Dongwhan Lee, John Yongmin Kim

**Affiliations:** Desert Orthopaedic Center, 2800 E Desert Inn Rd, Las Vegas, NV 89121 USA

**Keywords:** Cellular bone allograft, MAPC, Map3, rhBMP-2, Lumbar spine fusion

## Abstract

**Background:**

Both the map3 Cellular Allogeneic Bone Graft® and recombinant human bone morphogenetic protein 2 (rhBMP-2, Infuse®) were developed to provide an alternative to iliac crest autograft, thus eliminating the morbidity associated with its harvest. The recent literature concerning adverse events associated with the use of rhBMP-2, however, highlights the need for a safe and effective alternative. The multipotent adult progenitor cells (MAPC) found in map3 allograft may provide this alternative. The purpose of this study is to report 1-year outcomes of patients treated via anterior lumbar interbody fusion (ALIF) using either map3 Cellular Allogeneic Bone Graft or rhBMP-2 for bony fusion.

**Methods:**

This was a retrospective evaluation of 41 patients treated via ALIF with either map3 or rhBMP-2 in a polyetheretherketone cage with posterior stabilization at 1, 2, or 3 consecutive levels (L3-S1). Patients were equally divided between treatment groups. The Oswestry Disability Index (ODI) and visual analog scores (VAS) for pain were documented as part of the standard of care. An independent radiologist assessed bridging of bone, disc height, and lordosis. Primary outcome measures included radiographic analysis of fusion by plain film and CTs. Secondary clinical outcomes included visual analogue scale for neck and arm pain and low back disability index scores.

**Results:**

The overall fusion rate was 91%, with no significant difference between groups. Improvements in ODI and VAS were observed among all patients (*p* < 0.001), with no significant difference between groups for ODI (*p* = 0.966) or VAS (*p* = 0.251). There was no significant difference in terms of changes to disc height and lordosis between groups (p < 0.05). The rhBMP-2 group had increased post-operative complications when compared to the map3 group, but the low numbers precluded statistical analysis.

**Conclusion:**

Improvements in radiographic and clinical findings were observed in both treatment groups one-year postoperatively. Map3 allograft demonstrated equivalent fusion rates to rhBMP-2. A review of surgical supply costs at the treatment facility favored map3 allograft for the treatment of patients with DDD undergoing an ALIF in 1–3 levels compared to rhBMP-2. Further studies to evaluate long-term outcomes and post-operative complications are required.

## Background

Spine fusion is one of the most common procedures performed in spinal surgery with approximately 488,000 cases performed in the USA [1]. There are several surgical approaches available to achieve a solid union. Iliac crest bone graft (ICBG) is recognized as the “gold standard” against which all other graft materials are compared. Although the fusion rates and time to fusion are generally excellent for ICBG, increased operating time and donor-site morbidity are major concerns with the use of this graft type [2–4].

Avoiding the use of ICBG altogether has increased with the proliferation of reports indicating the effectiveness of additional graft options, such as bone marrow aspirate, local autogenous bone, allografts, synthetic materials, and recombinant human bone morphogenetic proteins (rhBMPs). While rhBMP-2 (Infuse®, Medtronic, Minneapolis, MN) has been used to facilitate fusion [5–7], there is a significant potential for adverse events (AE) [8, 9]. A critical review of published clinical studies observed an increased risk of retrograde ejaculation (RE) and other AE (e.g., inflammatory responses, heterotopic bone formation, radiculitis, osteolysis with cage/graft dislodgement/subsidence) for ALIF fusions utilizing BMP/INFUSE “on-label” [10–15]. Among the more worrisome complications is a potential increase in cancer risk [16]. Additionally, it has been noted that the use of rhBMP-2 does not enhance the fusion rate in stand-alone ALIF with femoral ring allografts (FRA’s) [14]. Investigators demonstrated a trend towards a higher nonunion rate with rhBMP-2 compared to a historical control ALIF using FRAs with autologous iliac crest bone graft. These results appear to be caused by the aggressive resorptive phase of allograft incorporation, which occurs prior to the osteoinduction phase.

Ideally, a bone graft would provide an osteoconductive matrix along with osteoinductive factors and osteogenic potential, while omitting the possible complications associated with autografts. While allografts offer some of the benefits of autograft without its limitations, they do not contain the viable cells necessary for osteogenesis. The addition of stem cell technology, among these being multipotent adult progenitor cells (MAPC), may add an important element to bone healing [17]. The role of these cells in bone healing and osteogenesis has been previously published, and the results point to an important clinical application that could benefit patients [18–20].

Furthermore, the bone is a highly vascular tissue [21], and successful remodeling depends on an adequate blood supply, which requires angiogenesis to re-establish the blood flow [21, 22]. This not only provides nutrients to the cells but allows inflammatory modulators and components necessary for regeneration to enter and leave the site of repair as needed. While bone morphogenetic proteins have been recognized as stimulating osteogenesis and increasing osteoblastic activity, there is still a need for blood vessels to support the new bone tissue [22]. In vitro testing has demonstrated the ability of MAPC-based cells to secrete vascular endothelial growth factor (VEGF), CXCL-5, and interleukin-8, all of which are important in stimulating angiogenesis [23].

With the numerous adverse events reported with the use of rhBMP-2, there is a compelling need for an alternative that can provide optimal conditions for bone fusion while limiting the potential for deleterious effects. A current product that is approved for use in lumbar fusion is map3® Cellular Allogeneic Bone Graft (RTI Surgical, Alachua, FL). This is composed of cortical-cancellous bone, demineralized bone matrix (DBM), and cryogenically preserved, viable MAPC-class cells. This retrospective investigation evaluated radiological outcomes in patients who had undergone a 1–3 level ALIF and received Infuse or map3 allograft.

## Methods

A consecutive series of 41 patients indicated for ALIF at 1–3 consecutive levels from L3–S1 were retrospectively analyzed. All patients were treated at a single surgical center. Only patients with clinical and radiographic evidence of degenerative lumbar spine disease were included. All fusion constructs were comprised of an anterior stand-alone interbody device (ROI-A; LDR, Austin, TX or PILLAR SA; Orthofix, Lewisville, TX) packed with map3 Cellular Allogeneic Bone Graft or rhBMP-2. Map3 is an allogeneic cancellous bone matrix, which also contains demineralized cortical bone and MAPC-based cells that have been derived from allograft bone marrow, isolated from other cells and cryopreserved. Both constituents are processed from the same donor, but are provided in separate containers. The implant is a combination of the scaffold and cells combined and are required to be used together. The rhBMP-2 also consists of two components—a recombinant human bone morphogenetic protein solution along with a carrier/scaffold for the bone morphogenetic protein solution. In addition, all patients received posterior stabilization, which consisted of segmental spinous process clamps (PrimaLOK SP; Osteomed, Addison, TX) or bilateral pedicle screw fixation (APEX; Zimmer, Warsaw, IN).

A total of 20 patients who received map3 allograft were retrospectively analyzed. These patients were similar with regard to diagnosis, number of fusion levels, smoking status, and comorbidity burden to 21 patients who had received rhBMP-2 (Table 1). An independent radiologist assessed the radiographs for fusion, which was defined as radiographic evidence of bridging across endplates, or bridging from endplates to interspace disc plugs. In specific cases where fusion was considered questionable, a CT scan was performed to confirm the presence of a bony fusion. Patients with previous failed fusion at the operative level, significant medical illness (e.g., active metastatic cancer or human immunodeficiency virus), or known conditions that would significantly inhibit bone healing (e.g., metabolic bone disease or uncontrolled diabetes) were excluded from enrollment. The Western Institutional Review Board approved this study (number 203609) and waived the requirement to obtain consent, as this was a purely retrospective study that followed the standard of care.

**Table 1:** Patient demographic characteristics by treatment group

	rhBMP-2	map3
	(*n* = 21)	(*n* = 20)
Segments treated	36	30
Male (*n*)	7	9
Female (*n*)	14	11
Age (years)	52.2 ± 10.3	53.9 ± 12.4
BMI	28.9 ± 5.5	28.7 ± 4.4
Obesity	7	5
Type II diabetes	2	4
Hypertensive	5	13
Current smokers	6	2
Former smokers	7	7

## Results

This level 3 retrospective, cohort comparison reports the results from a total of 41 patients, representing 66 treated segments at one study site. The average age at the time of surgery was 53 years, and the average BMI was 28.8. There was no significant difference between the two groups with respect to either BMI (*p* = 0.847) or age (*p* = 0.633). There were 16 males and 25 females. One-segment instrumented arthrodesis was performed in 20 patients, 2-segment instrumented arthrodesis was performed in 17 patients, and 3-segment instrumented arthrodesis was performed in 4 patients. Comorbidities included type-2 diabetes, obesity, smoking, and hypertension. Table 1 describes the patient demographics by treatment group.

### Clinical findings

**Fig. 1:**
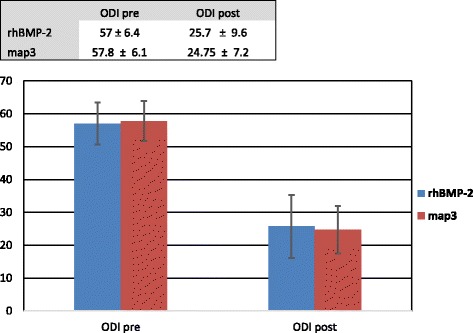
A repeated-measures ANOVA indicated that while patients significantly reduced their ODI score (*p* < 0.001), there was no difference in the change in ODI when compared between the patients that received either biologic (*p* = 0.966)

The changes in Oswestry Disability Index (ODI) are presented in Fig. 1. All patients studied reported improved function, regardless of the treatment group. The change in ODI was statistically significant for both treatment groups, with a mean improvement of 56% (*p* = < 0.001). However, there was no significant difference in ODI change between the map3 allograft and rhBMP-2 groups (*p* = 0.966).

**Fig. 2:**
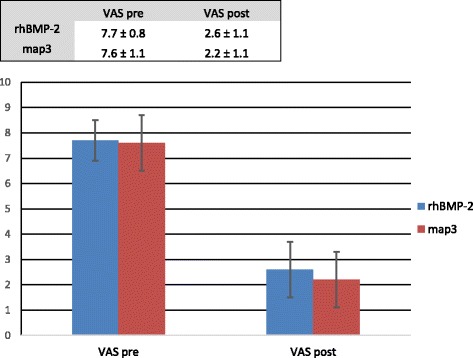
Similar to the results for ODI, a repeated-measures ANOVA indicated that the patients experienced a significant change in VAS (*p* < 0.001) but there was no difference in VAS when compared between groups. (*p* = 0.251)

The changes in VAS scores are presented in Fig. 2. Preoperative and postoperative VAS scores were recorded as numerical values 0 to 10. All patients recorded a positive change in postoperative self-reported pain. A comparison of pre and post-operative VAS scores revealed a significant decrease in pain for the combined groups, with a 67.9% mean improvement (*p* < 0.001). Similar to the ODI results, there was no significant difference between map3 allograft and rhBMP-2 (*p* = 0.251).

### Radiographic findings

An independent radiologist assessed bridging of bone, disc height, and lordosis. Fusion was defined as evidence of bridging across endplates, or bridging from endplates to interspace disc plugs. Radiographic analysis demonstrated a mean fusion rate of 91% for both groups at 12 months postoperatively. There was no statistical difference in segments fused when comparing map3 allograft and rh-BMP2 (*p* = 0.89). The arthrodesis rates, separated by the number of segments fused, are shown in Table 2. Changes in disc height and lordosis are presented in Table 3. The average disc height and lordosis increase was 1.70 and 3.14 mm, respectively, for map3 allograft. Similarly, the disc height and lordosis increase was 1.75 and 2.96 mm, respectively, for rhBMP-2. There was no significant difference in terms of changes to disc height and lordosis between map3 allograft and rhBMP-2 (*p* < .05). Additionally, there were no signs of gross instability, even among the pseudoarthrosis group that was provided anterior posterior instrumentation.

**Table 2:** Arthrodesis rates

	rhBMP-2		map3	
	# of individual sites	Fused (%)	# of individual sites	Fused (%)
1-segment arthrodesis	9	78	11	100
2-segment arthrodesis	18	100	16	81
3-segment arthrodesis	9	89	3	100
Total Segments	36	92	30	90

There was no difference in the proportion of patients who demonstrated radiographic evidence of fusion (Fisher’s exact test, *p* = 1)

**Table 3:** Changes in disc height and lordosis

	Disc height		Lordosis	
rhBMP-2	Pre	Post	Pre	Post
L5-S1	7.12	10.02	13.45	16.21
L4-L5	7.32	9.89	8.12	12.98
L3-L4	9.12	10.88	8.78	13.21
L2-L3	9.89	9.67	8.35	8.12
map3				
L5-S1	7.32	9.78	13.2	15.82
L4-L5	6.86	10.24	8.63	13.87
L3-L4	9.28	10.32	9.33	14.21
L2-L3	10.21	10.11	8.22	8.03

There was no significant difference in changes to disc height and lordosis between map3 allograft and rhBMP-2 (*p* ˂ .05)

**Fig. 3:**
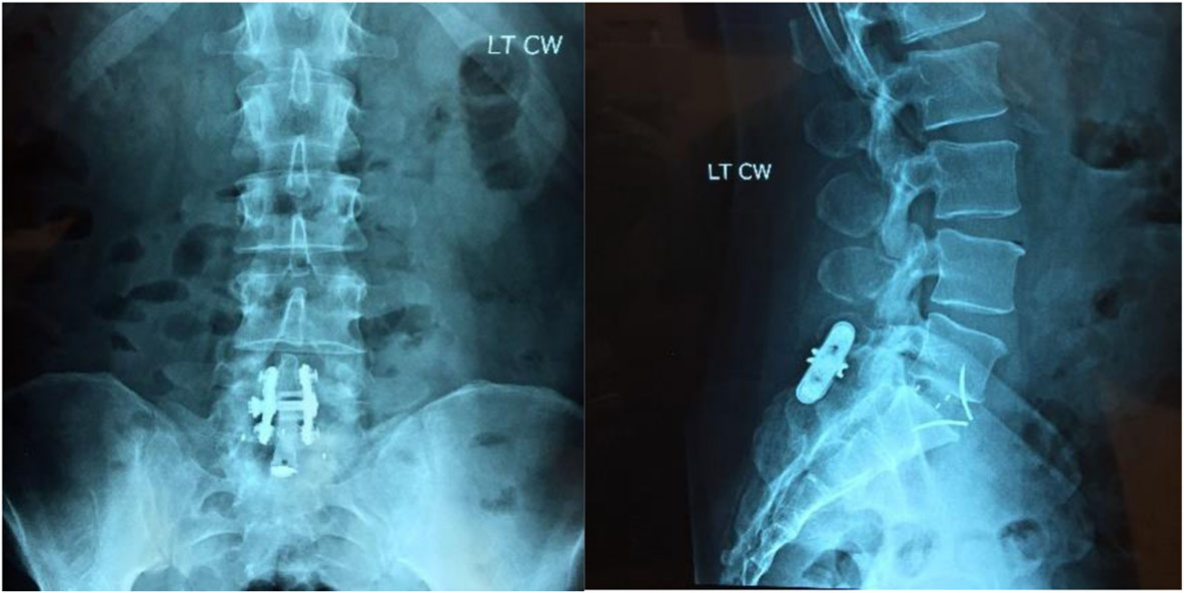
Twelve-month visit AP and lateral radiograph of subject who received 5cc’s of map3 Cellular Allogeneic Bone Graft

**Fig. 4:**
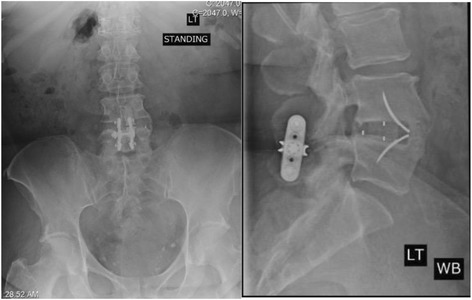
Twelve-month visit AP and lateral radiograph of subject who received 4.2mg of rhBMP2

At 1 year postoperatively, AP and lateral images demonstrate good restoration of the L5-S1 disc height with solid endplate incorporation of both the map3 allograft (Fig. 3) and rhBMP-2 (Fig. 4). New bone formation is observed anterior to the implants. There is no evidence of hardware loosening or PEEK implant subsidence noted. There was no motion of the fused segments observed in either the flexion or extension radiographs.

### Complications

Table 4 lists overall and individual postoperative complications. Complications occurred among 12 patients who received rhBMP-2 and 6 patients who received map3 allograft. There were no reports of intraoperative or major complications (e.g., death or neurological damage), or significant pain associated with sexual activities post operatively or retrograde ejaculation.

**Table 4:** Post-operative complications summary

	rhBMP-2	map3
Retroperitoneal hematoma	2	0
Posterior I&D	4	3
Postoperative radiculitis	8	2
Partial right foot drop	1	0
Epidural hematoma	1	1
Total	16	6

Two patients that underwent a fusion in the rhBMP-2 group experienced a retroperitoneal hematoma. Additionally, two patients in the map3 allograft group experienced postoperative radiculitis compared to eight patients in the rhBMP-2 group. One patient from each group experienced an epidural hematoma. There were seven total cases of posterior infection and drainage. Although a Fisher’s exact test demonstrated a strong trend towards a significant difference in the proportion of post-operative complications (*p* = 0.0578) between the treatment groups, this should be interpreted with some degree of caution due to the low numbers of patients and the high risk of a Type 2 error.

## Discussion

Achieving spinal fusion is one of the major endpoints for successful spine surgery. Although surgical techniques and technology have advanced, achieving fusion is still a major concern for the spine surgeon. Spinal pseudoarthrosis can be painful and lead to clinical failure.

Cellular bone matrices (CBMs) that contain live mesenchymal or MAPC stem cells are an adjunct and extender to achieve fusion. The utility of these CBMs to achieve spinal fusion is currently being investigated in numerous ongoing trials; however, to date, there are only three published retrospective studies evaluating CBMs and fusion in the spine. Kerr et al. (2011) reviewed 52 consecutive patients who underwent lumbar fusion with Osteocel at 1 or 2 contiguous levels and reported that solid arthrodesis was achieved in 92.3% of patients when used in lumbar interbody devices [24]. Similar results were published by Ammerman et al. (2013) who reviewed 23 patients undergoing MISS TLIF with MSC and noted a 91.3% fusion rate based on plain radiographs [25]. Tohmeh et al. (2012) had a 1-year follow-up on 40 patients who underwent an extreme lateral interbody fusion with an MSC-based allograft and reported a 90.2% fusion rate [26]. These fusion rates are comparable to that of the gold standard iliac crest bone graft. However, a criticism of at least one of these articles is that it was industry funded, while the other two articles did not disclose potential conflicts of interests. Therefore, the evidence-based literature is still meager. Although early results have been positive demonstrating equivalent, if not superior rates of fusion when compared to iliac crest bone graft, there is still a requirement of unbiased further investigation and study of CBMs.

The results of this paper are similar to the three published studies reporting fusion rates using CBMs in spine surgery patients [24–26]. However, this paper differs from these three published studies in that it compares rhBMP-2 surgical fusion results directly to map3 allograft, as well as restricts this comparison to ALIFs with posterior stabilization, thus eliminating a potential source of variability. There are no published CBM studies that examine solely ALIF with CBMs and posterior stabilization. Therefore, our results are a substantive addition to the current evidence on CBMs. Additionally, this study is devoid of any industry influence.

Findings from the study demonstrated that there was no clinical or statistical difference between the intraoperative biologics. In addition, there were no intraoperative or major complications. Favorable functional and clinical results were observed in the presence of common comorbidities such as obesity, smoking, and type-2 diabetes.

An allograft bone matrix containing both cancellous bone and demineralized cortical bone, as well as viable MAPCs offers a comprehensive osteoconductive, osteoinductive, and osteogenic product. MAPC-based technology is a specific stem cell that differs from other cells, e.g., MSC, currently available in the orthopedic market. MAPC-based cells have been shown to play an important role in not only bone formation, but immunomodulation and angiogenesis as well. In addition these cells have been shown to have a greater differentiation capability compared to MSC [27]. Furthermore, MAPC cells have been shown to have the capability to differentiate along an osteogenic lineage [28] as well as expressing the proteins essential for angiogenesis [23].

Although autograft from the iliac crest contains osteogenic, osteoconductive, and osteoinductive elements essential for the formation of new bone, there are concerns regarding the highly variable quality of the bone depending on age, metabolic abnormalities, and smoking history of the patient, as well as painful donor site morbidity issues [2–4], lack of quantity, and previous harvesting. Thus, there may be a reluctance from spine surgeons to use ICBG in spinal fusion given these issues. It is evident that the search continues among the spine surgeon community for a bone graft extender that has the efficacy of ICBG and rhBMP2, without the complications and morbidity associated with these grafts. In this study, using map3 allograft, the fusion rates were equivalent to rhBMP2 with fewer graft-related adverse events. Additionally, the fusion rates we have reported are comparable to what has been observed with ICBG. It is evident that map3 allograft is an appropriate adjunct to fusion in ALIFs.

Another factor to consider in the treatments in this study is that the benefit of cost savings per level with map3 allograft is significant and is an added advantage when compared to rhBMP-2. Map3 Cellular Allogeneic Bone Graft and rhBMP-2 intraoperative costs at the facility performing the study were compared to a national database that includes the average selling prices of cellular bone matrices (CBMs) and rhBMP-2 [29]. National hospital supplied data reports the costs of a 5cc CBM to be approximately 35% less compared to 4.2 mg of rhBMP-2 [25]. The savings found in the national database correlated with the findings in this study. Intraoperative biologic cost analysis revealed 35% cost savings in favor of map3 allograft for a single level segment, 40% for a 2-level and 45% cost savings for a 3-level segment compared to rhBMP-2. A spine surgeon’s costs per case, as well as the patient’s clinical outcome, are important variables in today’s healthcare environment. Map3 allograft allows the surgeon to maintain high fusion rates with notable cost savings, which is a favorable combination.

While overall post-operative complications were low, there was a strong trend towards increased complications in the rhBMP-2 group. The major difference between the treatment groups was the postoperative radiculitis demonstrated in the rhBMP-2 group. Theoretically, the rhBMP-2 group may have had more inflammation when compared to the map3 group. The post-operative radiculitis while transient did tend to resolve over time, necessitating medication and further treatment.

The limitations of this study can be summarized in the small study population as well as the retrospective nature of it. The sample size did not have the power to determine statistically significant differences between groups if they do exist. The uncontrolled retrospective reviews allow for the inclusion of variables that might be confounding or produce bias. Coincidentally, patients in both treatment groups were similar in demographic and medical make up, which is a benefit in the analysis.

While the limited peer-reviewed literature demonstrates the efficacy and fusion outcomes of MSC, additional studies are still warranted. Ideally, large prospective randomized controlled trials would be completed; however, these types of studies in surgical spine patients are rare given the costs, enormity of the undertaking, and potential for ethical controversy.

## Conclusion

In conclusion, independent radiologic assessment of the anterior interbody fusion with posterior stabilization demonstrated bridging bone and fusion in 91% of segments overall with no statistical difference between intraoperative biologics. There were no intraoperative or major complications. Favorable functional and clinical results were similarly observed in both groups. Map3 allograft appears to be a safe and effective bone graft extender in lumbar fusion. Further studies are required for CBMs provided the relative paucity of clinical, peer-reviewed articles and therefore, further define the clinical benefit and risk profile.

## Abbreviations

A/P: Anterior/posterior
BGS: Bone graft substitute
BMI: Body mass index
BMP: Bone morphogenetic protein
CBM: Cellular bone matrix
CT: Computed tomography
DBM: Demineralized bone matrices
DDD: Degenerative disc disease
ICBG: Iliac crest bone graft
MAPC: Multipotent adult progenitor cell
MSC: Mesenchymal stem cell
PLF: Posterolateral fusions
SD: Standard deviation
